# Metformin may alter the course of Leber’s hereditary optic neuropathy: a case report

**DOI:** 10.3389/fmed.2025.1609941

**Published:** 2025-08-19

**Authors:** Shenoda Abd Elmaseh, Danielle A. Gauthier, Maryam Golmohammadi, Nutsa Pargalava, Valerio Carelli, Alfredo A. Sadun

**Affiliations:** ^1^Department of Ophthalmology, David Geffen School of Medicine, Los Angeles, CA, United States; ^2^Doheny Eye Institute, Los Angeles, CA, United States; ^3^Department of Biomedical and Neuromotor Sciences, University of Bologna, Bologna, Italy; ^4^Programma di Neurogenetica, IRCCS Istituto di Scienze Neurologiche di Bologna, Bologna, Italy

**Keywords:** metformin, LHON, idebenone, NAD+, vision Loss, mitochondrial dysfunction

## Abstract

Leber’s hereditary optic neuropathy (LHON) is a rare inherited mitochondrial disease caused by variants in mitochondrial DNA (mtDNA) transmitted exclusively through the maternal line. The disease predominantly affects young males and is characterized by progressive bilateral vision loss. Idebenone, a well-studied drug, modestly enhances the mitochondrial function and visual acuity in many patients with LHON. In this study, we report the case of a 48-year-old woman diagnosed with LHON (m.11778G>A/*MT-ND4*) and type 2 diabetes mellitus who experienced visual field improvement following metformin treatment after 26 months of progressive vision loss unresponsive to idebenone, nicotinamide adenine dinucleotide (NAD+), and hormone replacement therapy (HRT). Our findings offer an intriguing perspective on LHON management but require more investigations, particularly on the molecular effects of metformin on the mitochondrial function in LHON patients.

## Graphical abstract

Metformin-induced visual field improvement was observed in a patient affected by Leber hereditary optic neuropathy (LHON) with concurrent type 2 diabetes mellitus (DM II). Prior treatments—including idebenone, NAD+, and hormone replacement therapy (HRT)—failed to show efficacy in this LHON-affected case (m.11778G>a/*MT-ND4*). DM II, type 2 diabetes mellitus; ROS, reactive oxygen species; HRT, hormone replacement therapy. Figure was created using BioRender. Source: Sadun A. (2025). Available online at: https://BioRender.com/uqb2o65.
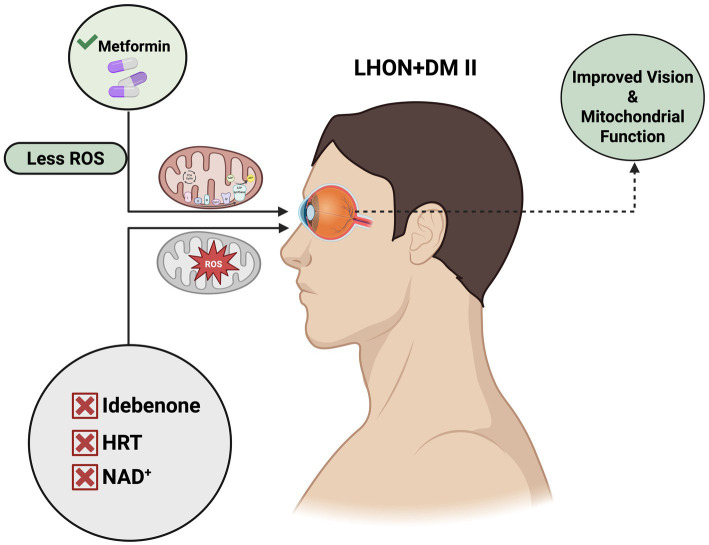

## 1 Introduction

Leber’s hereditary optic neuropathy (LHON) is a maternally inherited mitochondrial disorder caused by missense point mutations in mitochondrial DNA (mtDNA) genes that encode subunits of respiratory complex I in the electron transport chain (ETC) (1, 2). The disease typically presents as bilateral, painless vision loss, with one eye usually affected first, followed by the second within 4 to 8 weeks (2). LHON predominantly affects young males aged 12 to 30 years, with a male-to-female ratio of approximately 5:1. Although the disease is most common in this demographic, it can occur at any age, with approximately 10% of cases reported in individuals over 50 years of age (3). Dysfunction of mitochondrial complex I (NADH ubiquinone oxidoreductase), caused by the three most common mtDNA mutations—m.11778G>A in *MT-ND4*, m.3460G>A in *MT-ND1*, and m.14484T>C in *MT-ND6*—disrupts energy production and leads to excessive generation of reactive oxygen species (ROS). These factors are critical to retinal ganglion cell (RGC) health and LHON pathogenesis (4, 5). Currently, idebenone—a quinone analog that bypasses complex I, facilitates electron transfer directly to complex III, and exhibits antioxidant properties—is the only EMA-approved LHON treatment. Although idebenone is the standard of care for LHON in Europe, it has not received regulatory approval in the United States (6).

**Figure 1:**
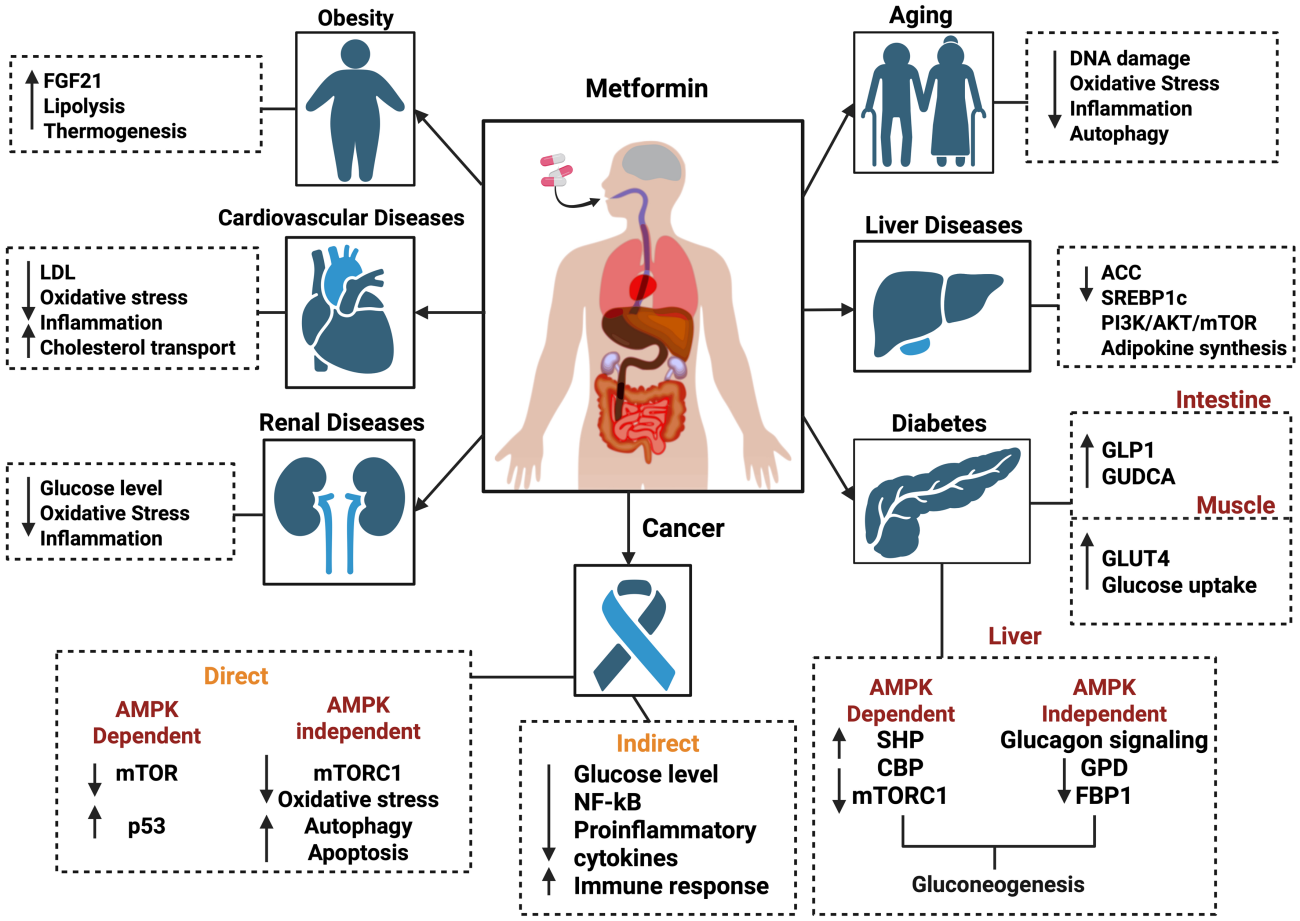
Schematic view of various molecular effects of metformin on different diseases, including obesity, cardiovascular diseases, renal diseases, cancer, diabetes, liver diseases, and aging. The figure was created in BioRender. Source: Sadun A. (2025). Available online at: https://BioRender.com/j88p452.

Metformin, a biguanide derivative, is a widely prescribed medication for type 2 diabetes (DM II) and has been in clinical use for nearly a century (5). Beyond its well-known role in glycemic control, recent studies have highlighted the strong therapeutic potential of metformin against various diseases, including cancer (7), cardiovascular disease (8), liver disorders, obesity (9), neurodegenerative diseases (10), and renal disorders (11, 12) (Figure 1). Remarkably, metformin has been associated with lactic acidosis (13), attributed not only to its interference with mitochondrial respiration at the complex I site (14) but also to its activation of mitochondrial biogenesis (15). This compensatory mechanism may improve mitochondrial function (16).

Here, we present an LHON-affected case with visual field improvement following metformin treatment who was unresponsive to other therapeutic interventions—including idebenone, NAD+, and hormone replacement therapy (HRT)—and evaluate metformin’s potential contribution to the recovery of visual function.

## 2 Case presentation

A 48-year-old woman presented to the ophthalmology clinic with decreased visual acuity, generalized central visual field depression, and impaired color vision in both eyes (OU). She reported initial vision deterioration in her left eye (OS) on 30 August 2021, followed by the involvement of right eye (OD) a few days later. Her medical history was unremarkable, except for perimenopause and maternally inherited m.11778G>A/*MT-ND1* mutation associated with LHON.

**Figure 2:**
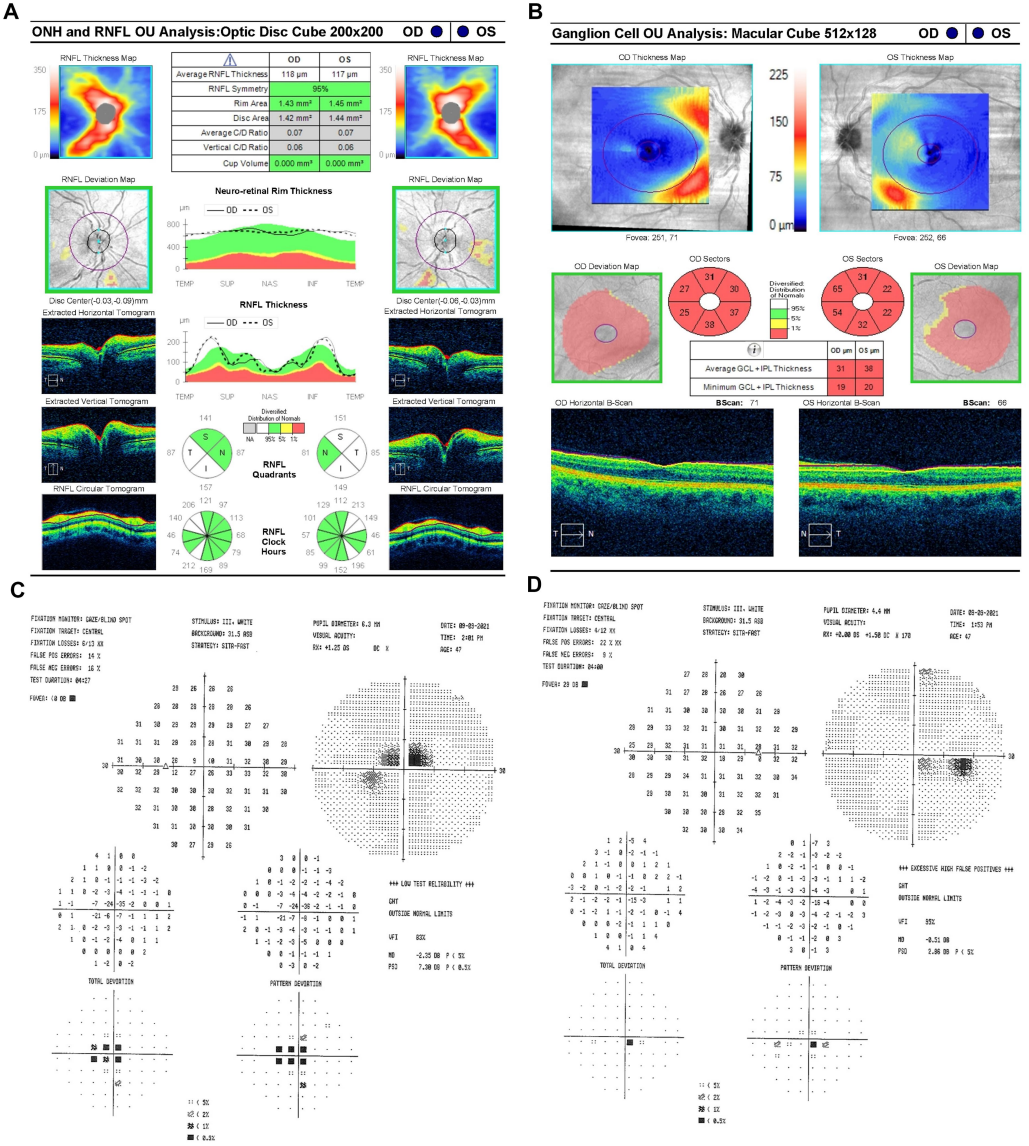
**(A)** Zeiss Cirrus OCT of the ONH and RNFL OU analysis: optic disc cube 200 × 200 scan showing a standard bilateral average RNFL thickness on September 9, 2021. **(B)** Zeiss Cirrus ganglion cell OU analysis: macular cube 512 × 128 scan showing diffuse GCL thinning and swelling on the same date. **(C)** Zeiss single-field analysis: central 30-2 HVF demonstrating the formation of a central scotoma OU, with standard MD values of −2.35 dB in OS **(D)** and −0.5 dB in OD on 9 September 2021. OCT, optical coherence tomography; ONH, optic nerve head; RNFL, retinal nerve fiber layer; GCL, ganglion cell layer; HVF, Humphrey visual field; MD, mean deviation; OU, both eyes; OS, left eye; OD, right eye.

On 9 September 2021 (10 days after symptom onset), carrier conversion was confirmed through clinical examination, showcasing classical LHON symptomatology of impaired color vision, reduced retinal nerve fiber layer (RNFL) thickness, and visual field (VF) defects. Best-corrected visual acuity (BCVA) was 20/80 in the OD and 20/100 OS. Pupils measured 4 mm bilaterally, with no relative afferent pupillary defect (RAPD). Intraocular pressure (IOP) measured 19 mmHg OU. Her neurological, sensory, and motor assessments were normal. The anterior segment and external ocular examinations were also normal. Ishihara color test scores were 10/14 OD and 3/14 OS, reflecting greater color vision impairment in OS. Optical coherence tomography (OCT) scans were performed using a Zeiss Cirrus HD-OCT system (Carl Zeiss Meditec, Inc., Dublin, California, United States) to measure structural changes in RNFL. OCT showed a normal average RNFL thickness of 118 μm OD and 117 μm OS with evidence of RNFL swelling, particularly in the temporal-inferior sectors (Figure 2A). Diffuse thinning of the ganglion cell layer (GCL) was observed, indicative of RGC loss (Figure 2B). Fundus examination and OCT showed no evidence of diabetic retinopathy at any point during follow-up. Standard achromatic 30-2 perimetry tests were performed using a Humphrey VF Analyzer (Carl Zeiss Meditec, Inc., Dublin, California, United States) to measure functional vision changes. Humphrey visual field (HVF) testing revealed central scotomas in OU, with a standard mean deviation (MD) of −0.5 dB OD (Figure 2C) and −2.35 dB OS (Figure 2D). Idebenone treatment was initiated at a dose of 300 mg orally three times daily after the confirmation of LHON.

**Figure 3:**
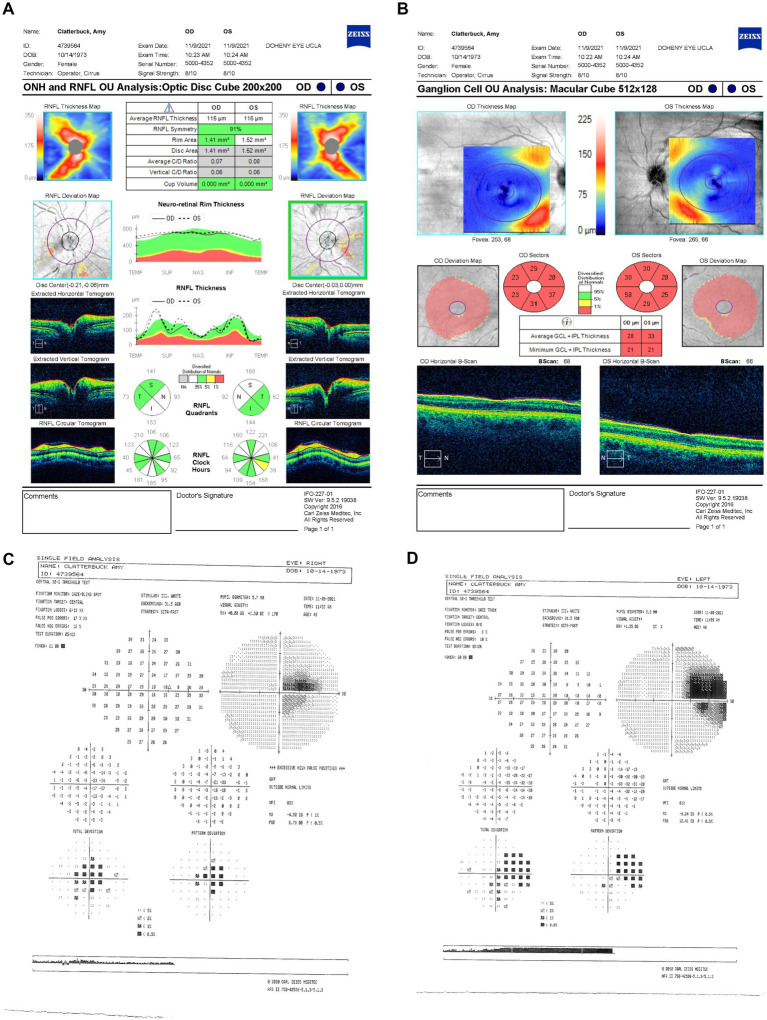
**(A)** Zeiss Cirrus OCT of the ONH and RNFL OU analysis: optic disc cube 200 × 200 scan showing a standard bilateral average RNFL thickness on 9 November 2021. **(B)** Zeiss Cirrus ganglion cell OU analysis: macular cube 512 × 128 scan showing diffuse GCL thinning and swelling on the same date **(C)** Zeiss single-field analysis: central 30-2 HVF illustrating the formation of a cecocentral scotoma, with a standard MD of −4.5 dB OS **(D)** and continued growth of the central scotoma, with a standard MD of −9.24 dB OD on 9 November 2021. OCT, optical coherence tomography; ONH, optic nerve head; RNFL, retinal nerve fiber layer; GCL, ganglion cell layer; HVF, Humphrey visual field; MD, mean deviation; OS, left eye; OD, right eye.

Her follow-up appointment on 9 November 2021 (2.5 months since LHON onset) showed persistent visual deterioration. Her BCVA declined to 20/250 −2 OD and 20/200 OS. Ishihara color tests and IOPs remained unchanged, with no RAPD. OCT showed an increased average RNFL thickness of 115 μm OD and 116 μm OS with ongoing temporal-inferior RNFL swelling (Figure 3A) and progressive diffuse GCL thinning (Figure 3B). HVF indicated further expansion of the central scotoma in OD with a MD of −9.24 dB (Figure 3C) and the development of a cecocentral scotoma in OS of −4.5 dB (Figure 3D).

**Figure 4:**
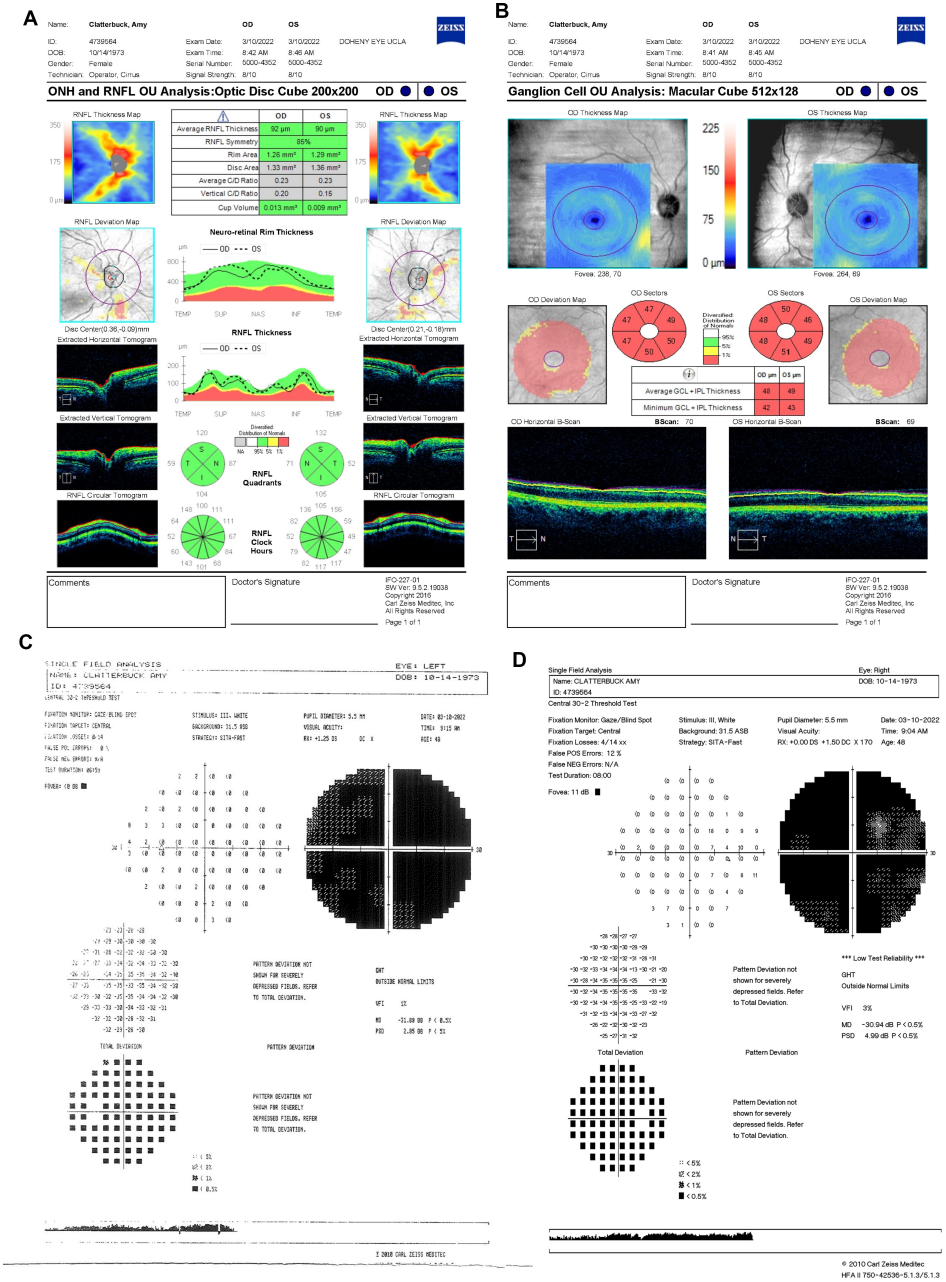
**(A)** Zeiss Cirrus OCT of the ONH and RNFL OU analysis: optic disc cube 200 × 200 scan showing a standard bilateral average RNFL thickness on 10 March 2022. **(B)** Zeiss Cirrus ganglion Cell OU analysis: macular cube 512 × 128 scan showing diffuse GCL thinning and swelling on the same date. **(C)** Zeiss single field analysis: central 30-2 HVF illustrating the formation of a cecocentral scotoma, with a standard MD of −31.89 dB OS **(D)** and continued growth of the central scotoma, with a standard MD of −30.94 dB OD on 10 March 2022. OCT, optical coherence tomography; ONH, optic nerve head; RNFL, retinal nerve fiber layer; GCL, ganglion cell layer; HVF, Humphrey visual field; MD, mean deviation; OS, left eye; OD, right eye.

Progressive visual loss was confirmed on 10 March 2022 (6.5 months from LHON onset), with suspected poor response to idebenone. BCVA had declined to 20/800 OD and counting fingers (CF) at three feet OS. Based on prior evidence of estrogen’s neuroprotective role in LHON (17, 18), the patient started HRT. She was unable to complete the Ishihara color test. OCT showed further RGC loss with RNFL thickness decreasing to 92 μm OD and 90 μm OS (Figure 4A) and persistent bilateral GCL thinning (Figure 4B). HVF demonstrated profound generalized visual field depression bilaterally with MD values of −30.94 dB OD (Figure 4C) and −31.89 dB OS (Figure 4D).

**Figure 5:**
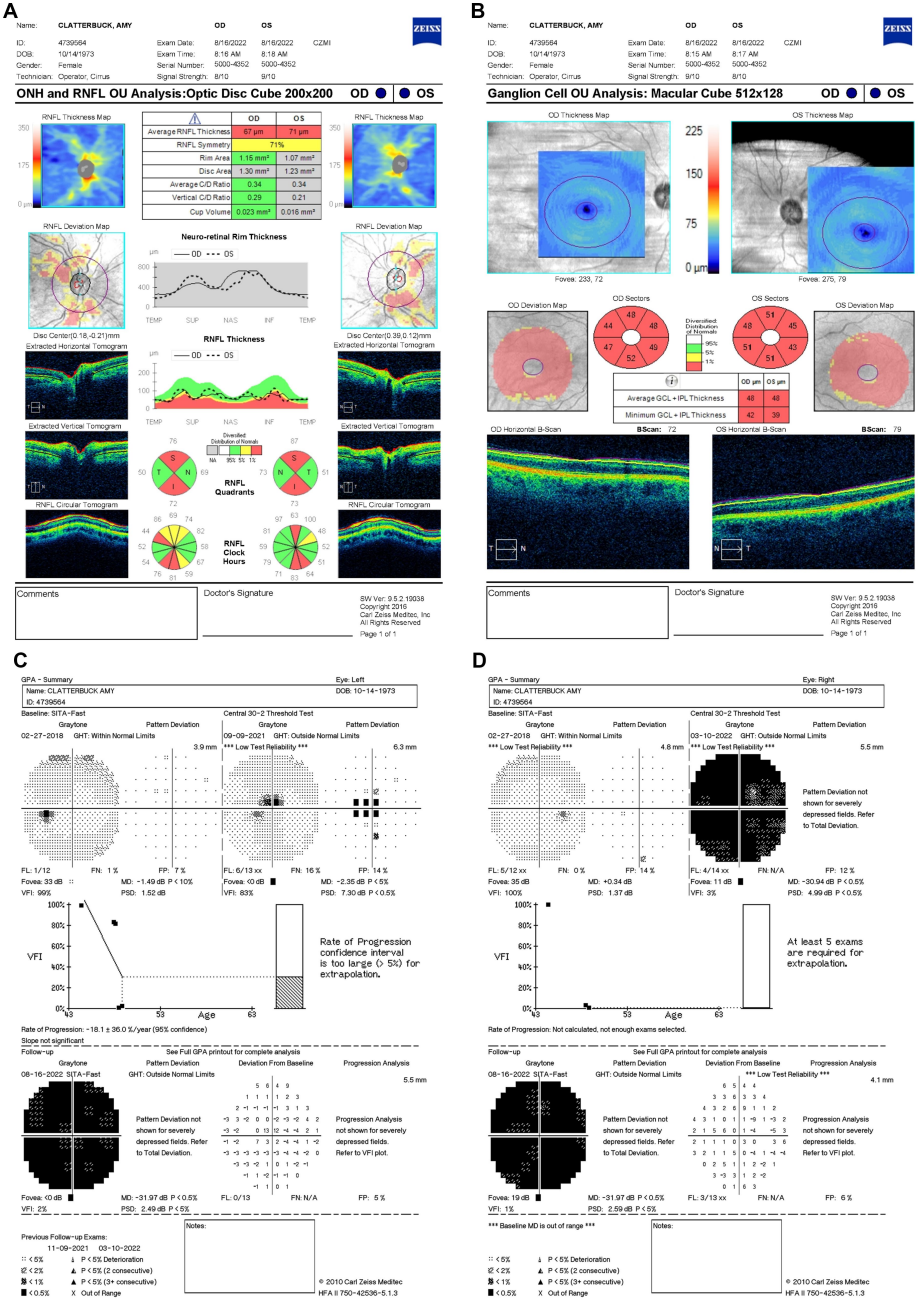
**(A)** Zeiss Cirrus OCT of the ONH and RNFL OU analysis: optic disc cube 200 × 200 scan showing a decreased standard bilateral average RNFL thickness **(B)** and diffuse GCL thinning and swelling on 16 August 2022. **(B)** Zeiss Cirrus ganglion cell OU analysis: macular cube 512 × 128 showing diffused GCL thinning and swelling on 16 August 2022. **(C,D)** Zeiss single-field analysis: central 30-2 HVF demonstrating profound central general depression, with a standard MD of −31.97 dB in both eyes on 16 August 2022. OCT, optical coherence tomography; ONH, optic nerve head; RNFL, retinal nerve fiber layer; OU, both eyes; GCL, ganglion cell layer; HVF, Humphrey visual field; MD, mean deviation.

**Figure 6:**
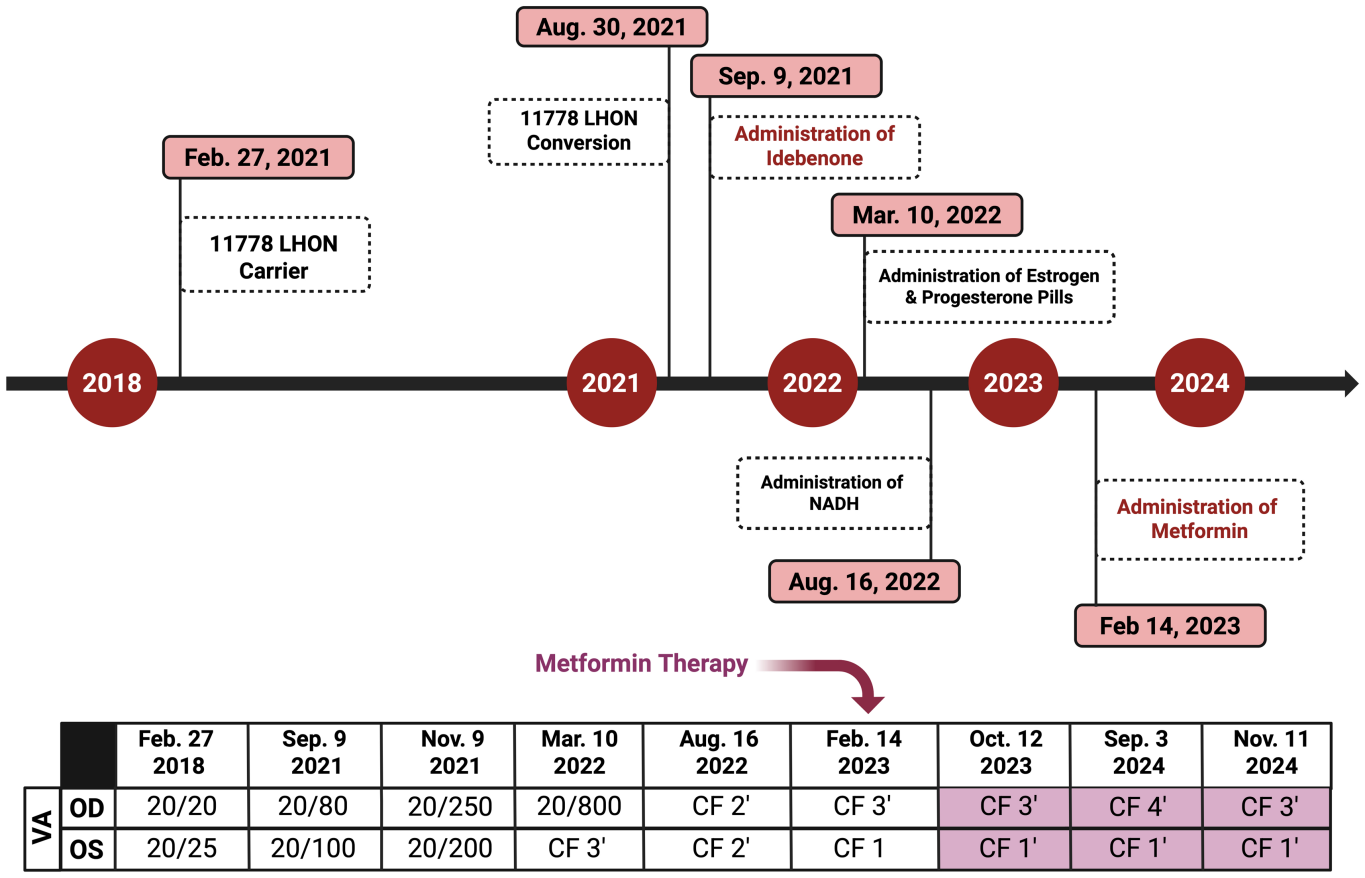
Timeline of key clinical events, medication interventions, and visual acuity (VA) changes in the affected LHON patient. The timeline illustrates the progression of vision loss, initiation of treatments, and subsequent changes in the VA testing (Snellen chart). The figure was created in BioRender. Source: Sadun A. (2025). Available online at: https://BioRender.com/v2qx9ed.

By 16 August 2022 (11.5 months after LHON onset), her BCVA had decreased to CF at two feet OU. Her OCT showed significant RNFL thinning of 55 μm OD and 57 μm OS, predominantly in the superior and inferior quadrants (Figure 5A), with further GCL thinning (Figure 5B). Similarly, HVF revealed worsening central scotoma with severe field loss with MD values of −31.97 dB OU (Figures 5C,D). Due to the lack of response to idebenone and HRT, the patient was prescribed an alternative 300 mg of NAD+ treatment three times daily (Figure 6).

**Figure 7:**
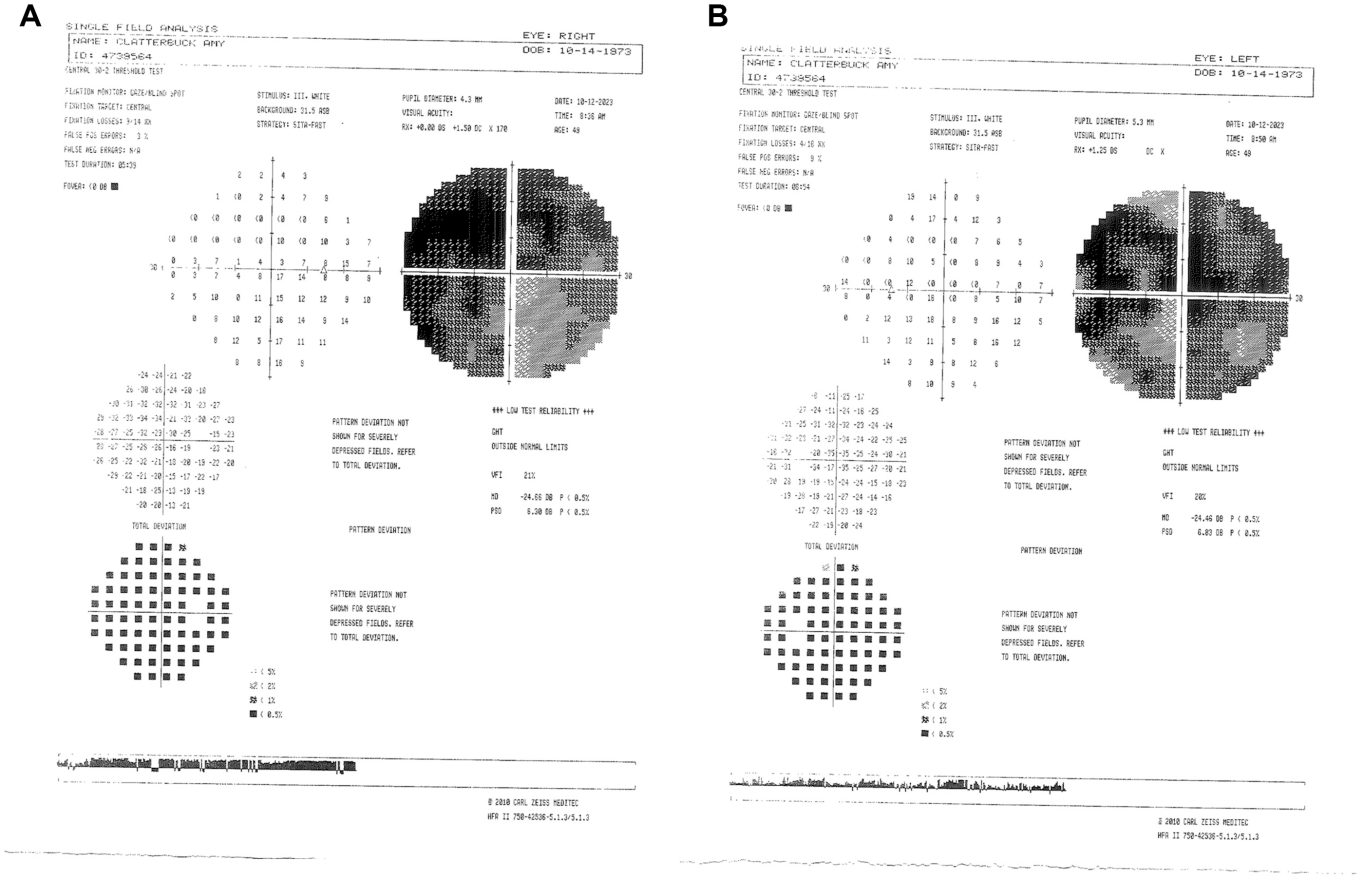
**(A)** Zeiss single-field analysis: central 30-2 HVF demonstrating profound central general depression, with a standard MD of −24.46 dB OS **(B)** and −24.56 dB OD on 11 November 2024. HVF, Humphrey visual field; MD, mean deviation; OS, left eye; OD, right eye.

On 14 February 2023 (17.5 months following LHON onset), the patient was diagnosed with DM II and subsequently was prescribed 500 mg of metformin once daily for glycemic control (Figure 6). About nine months after starting the metformin treatment course, she subjectively reported improved visual function. She could recognize faces at close distance, which she had been unable to do before therapy. In her follow-up on 11 November 2024 (26 months following LHON onset), her BCVA deteriorated to CF at three feet OD and improved to CF at one foot OS. The HVF showed an improvement in central general depression, with mean deviation (MD) values improving to −24.56 dB OD (Figure 7A) and −24.46 dB OS (Figure 7B) from February 2023, with standard MD values of −29.54 dB OD and −29.86 dB OS (Figure 6). At the time of metformin initiation, the patient continued idebenone, NAD+, and HRT. While spontaneous recovery occurs in a few m.11778G>A/*MT-ND4* cases, the timing and correlation with metformin suggest a potential therapeutic effect, possibly in combination with metabolic improvements and the other treatments. The patient reported good adherence to all prescribed therapies and did not experience any adverse events throughout the treatment period. She reported severe emotional and functional distress following the onset of vision loss that impaired her daily activities. After the initiation of metformin, she subjectively reported improvements, such as the ability to recognize faces at near distances.

## 3 Discussion

**Figure 8:**
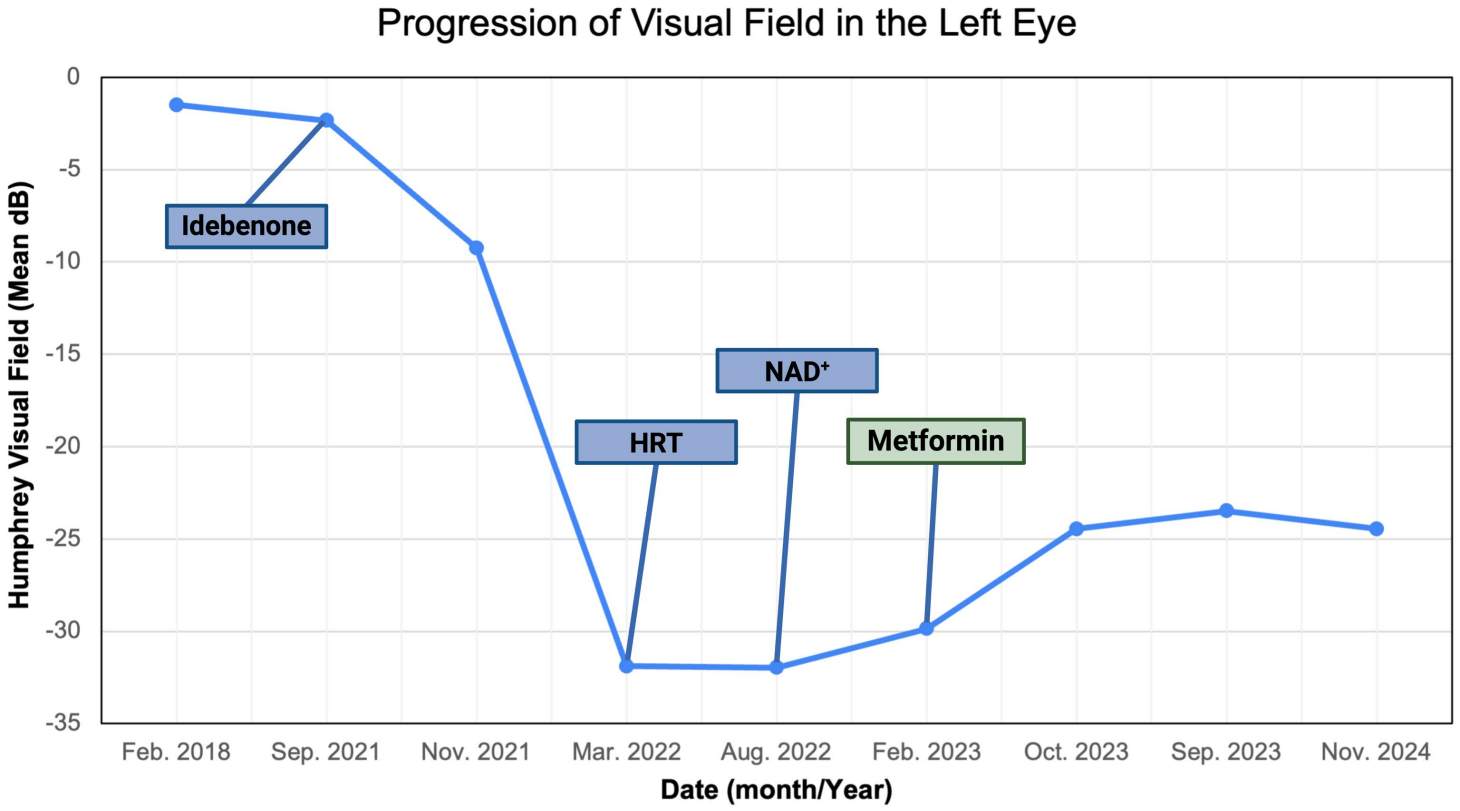
Zeiss single-field analysis: central 30-2 HVF OS from 27 February 2018 to 11 November 2024. The graph depicts visual field recovery over 2 years from the lowest standard MD of −31.89 to −24.46. HVF, Humphrey visual field; MD, mean deviation; OS, left eye. The figure was created in BioRender. Source: Sadun A. (2025). Available online at: https://BioRender.com/sdyj4x2.

**Figure 9:**
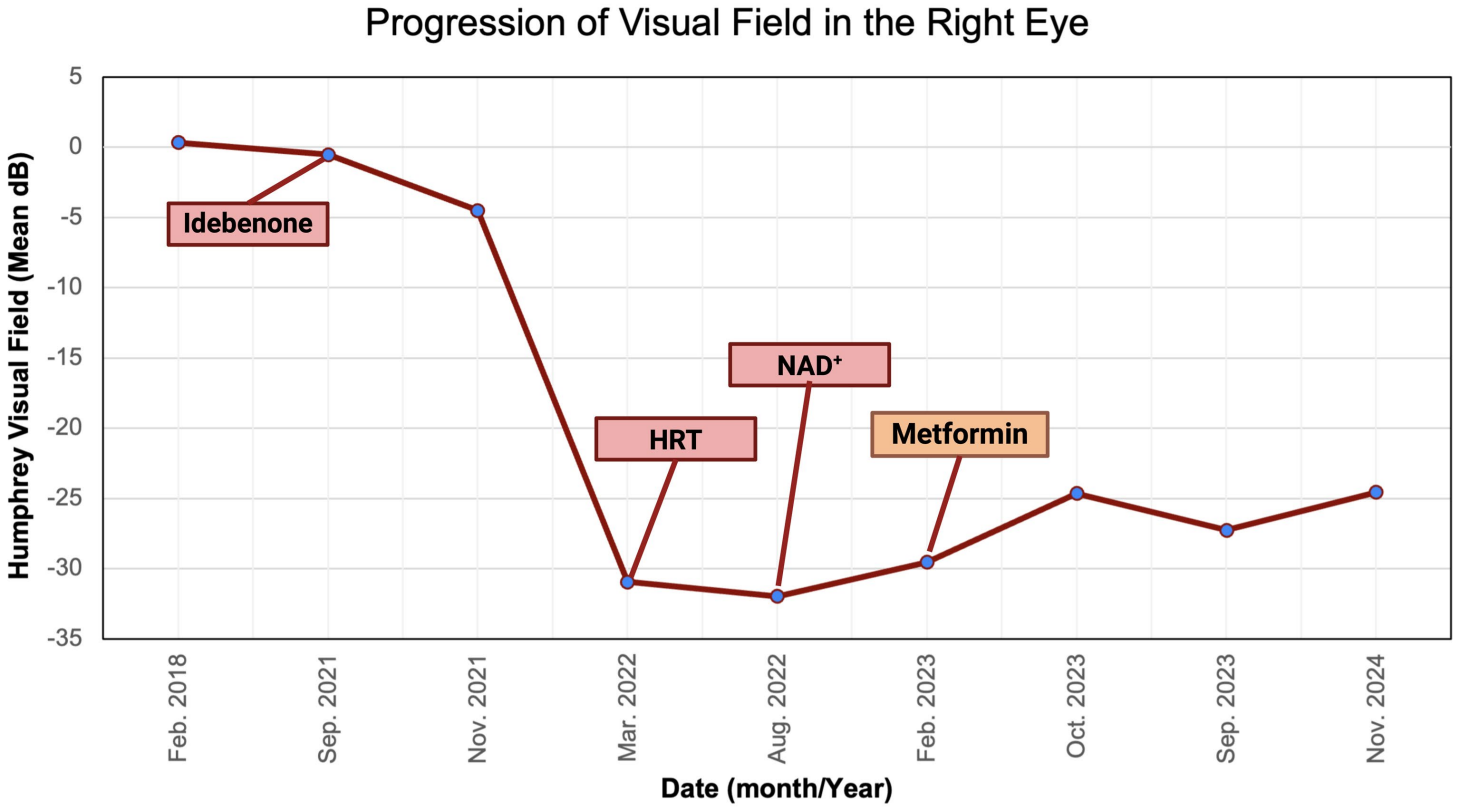
Zeiss single-field analysis: central 30-2 HVF OD from 27 February 2018 to 11 November 2024. The graph depicts visual field recovery over 2 years from the lowest standard MD of −30.94 to −24.66. HVF, Humphrey visual field; MD, mean deviation; OD, right eye. The figure was created in BioRender. Source: Sadun A. (2025). Available online at: https://BioRender.com/b2h0k6y.

We described a case of visual improvement in a postmenopausal woman with LHON and DM II following metformin administration. The treatment with metformin was for managing her DM II. It was initiated 17.5 months after the onset of vision loss and the lack of improvement with the other therapies (i.e., idebenone, HRT, and NAD+). Functional improvement corroborated a possible LHON metformin benefit indicated by a decrease in standard MD on HVF from −31 dB to −25 dB OD and from −32 dB to −25 dB OS (Figures 8, 9). While there are reported spontaneous remissions in some LHON patients (19), the relationship of metformin therapy with visual improvement supports causality, making further investigation into its mechanisms worthwhile. The improvement observed 26 months post-onset shows that retinal ganglion cells may remain viable but are dysfunctional for longer than previously believed, and therefore, reconsideration of the therapeutic window in LHON is warranted (1, 19).

Metformin has been increasingly recognized for its effects on the longevity of life, aside from its traditional indication as the first-line treatment for DM II (20). Its multiple effects on oxidative stress, inflammation, and neuroprotection gave us reason to be intrigued by her incidental visual improvement in our LHON-affected case (21, 22). This case report introduces a potential therapeutic direction for LHON, the mitochondrial disorder characterized by RGC degeneration and progressive vision loss. It highlights the general implications of metformin’s mitochondrial-modulating effects. While the underlying mechanisms of metformin’s effects on LHON remain unclear, some hypotheses may explain its potential benefits to mitochondrial function in the context of mitochondrial dynamics, biogenesis, and mitophagy (23).

A possible benefit of metformin is its ability to influence mitochondrial biogenesis by decreasing excess ROS production and subsequent RGC loss, both of which are predominant consequences of LHON pathogenesis. When metformin binds onto a specific complex I site (24, 25), it selectively inhibits complex I activity to prevent a reverse-electron flux (26–28). This inhibition suppresses electron transfer from NADH to electron acceptors to generate water and replenish NAD+ (29), resulting in lowered adenosine triphosphate (ATP) intracellular levels and higher adenosine diphosphate (ADP) and adenosine monophosphate (AMP) intracellular concentrations. As a result, the mtDNA copy number increases, creating a neuroprotective effect (30, 31). Concurrently, adenosine 5′-monophosphate-activated protein kinase (AMPK) is activated, acting as a metabolic pathway regulator (32, 33) known for inducing autophagy and decreasing ROS overproduction. As oxidative stress reduces, it creates a protective microenvironment for neuronal preservation (34–36). Therefore, reducing ROS production may affect neuronal function. As a result, it may provide some neuroprotection and enhance neuron survival (37, 38).

On the other hand, metformin-induced mitophagy may enhance cellular function by restoring a healthy mitochondrial phenotype in LHON. The accumulation of damaged mitochondria could exacerbate the ETC dysfunction caused by LHON, leading to oxidative stress and RGC death (39). Multiple studies have demonstrated that a homeostatic program controls mitochondrial production and balances mitophagy and mitobiogenesis (40). This program is regulated by sensing oxidative phosphorylation efficiency and demand (41), which is tightly related to ROS production and retrograde signaling systems collaborating between mtDNA and the nuclear genome (42). In LHON, this connection may lead to very different outcomes, as exemplified by the asymptomatic LHON carriers when compared with LHON-affected patients. The efficiency of mitochondrial biogenesis controls the cellular fate (17, 43) regarding autophagy and mitophagy (44). Metformin’s effects on autophagy are contradictory. While it may induce autophagy via various AMPK-related signaling pathways in some context, it could also inhibit excessive autophagy and apoptosis, thereby preserving RGCs (12, 39, 45).

Another hypothesis indicates that metformin may lower inflammation in LHON patients. Although mitochondrial dysfunction contributes to neuroinflammation, LHON has traditionally been regarded as non-inflammatory due to the absence of optic disc leakage on fluorescein angiography. However, it is speculated that since metformin downregulates the expression of pro-inflammatory cytokines, such as TNF-α and IL-6, via NF-𝜅B inhibition and contributes to the suppression of aging-related inflammatory pathways (46–48), similar benefits may be seen in LHON. Metformin directly affects peripheral blood mononuclear cells by entering through the human organic cation transporter type 1, modulating the inflammatory response and mitochondrial dynamics. Metformin further suppresses inflammation and raises the apoptosis threshold by reducing insulin levels, IGF-1, and mTOR signaling (23, 49). Despite these compelling studies, the possible pathophysiologic influence of metformin on inflammation in LHON remains unclear.

While this case raises intriguing ideas of possible metformin benefit, some limitations must be considered. The data are based on one case report, and the possibility of spontaneous remission cannot be excluded, particularly given the variable prognosis of the m.11778G>A/*MT-ND4* mutation. While the integration of metformin coincided with some visual field improvement, the patient could be a late responder to idebenone, as it takes up to 2 years to notice the full benefit. Other limitations involve the combination of all therapies leading to a synergistic effect on biogenesis and mitophagy. For instance, the interaction of metformin with hormonal effects, given the postmenopausal status of the patient, could have contributed to the improvement. Although idebenone, NAD+, and HRT had shown no immediate benefits, they may have contributed to a delayed or synergistic effect with metformin. Earlier reports have shown that estrogen has neuroprotective effects in LHON (17). The benefit of metformin could be synergistic or independent, perhaps augmented by its metabolic effects in the context of DM II. This result underlines the need to ascertain the interaction of metformin with sex hormones in LHON and other mitochondrial disorders.

Finally, the specific molecular pathways linking metformin with visual restoration are speculative without experimental validation in LHON models. The confounding factor that the patient has DM II concurrently raises the possibility that metformin glycemic control indirectly influences mitochondrial function and visual restoration. We cannot exclude the fact that improved glycemic control from metformin contributed indirectly to improved mitochondrial function and visual recovery. Future studies would be enhanced by longitudinal clinical trials with larger groups and molecular analyses to replicate these findings, including molecular or biochemical measurements, such as ROS levels, AMP-activated protein kinase (AMPK) activation, and mtDNA copy number in RGCs. Finally, we recognize inherent HVF and visual acuity measurement variability in profound vision loss and a possible learning effect for VF testing. More objective visual function and recovery visual biomarkers, such as OCT-based metrics, electrophysiology, and photopic negative response, should be incorporated in future research.

## 4 Conclusion

This case highlights the potential role of metformin in promoting visual function in a patient with LHON and DM II. Although the exact mechanisms remain unclear, metformin’s inhibitory effects on complex I and the reduction of oxidative stress may alter the course of LHON type II patients. Additional studies on the molecular level are needed to explain metformin’s role and confirm its protective role in LHON patients. Moreover, a clinical trial with a larger sample size may help confirm this benefit.

## Data Availability

The datasets generated during and/or analyzed during the current study are not publicly available, but are available from the corresponding author on reasonable request.

## Glossary

LHON: Leber’s hereditary optic neuropathy
mtDNA: Mitochondrial DNA
ETC: Electron transport chain
RGCs: Retinal ganglion cells
ROS: Reactive oxygen species
DM II: Type 2 diabetes mellitus
RNFL: Retinal nerve fiber layer
VF: Visual field
OU: Both eyes
OS: Left eye
OD: Right eye
BCVA: Best corrected visual acuity
RAPD: Rapid afferent pupillary defect
IOP: Intraocular pressure
OCT: Optical coherence tomography
GCL: Ganglion cell layer
MD: Mean deviation
HRT: Hormone replacement therapy
ATP: Adenosine triphosphate
ADP: Adenosine diphosphate
AMP: Adenosine monophosphate
AMPK: Adenosine 5′-monophosphate-activated protein kinase
